# Subcellular Targeting of the *Euplotes raikovi* Kinase *Er*-MAPK1, as Revealed by Expression in Different Cell Systems

**DOI:** 10.3389/fcell.2019.00244

**Published:** 2019-10-17

**Authors:** Annalisa Candelori, Takaharu G. Yamamoto, Masaaki Iwamoto, Maura Montani, Augusto Amici, Adriana Vallesi

**Affiliations:** ^1^School of Biosciences and Veterinary Medicine, University of Camerino, Camerino, Italy; ^2^Advanced ICT Research Institute, National Institute of Information and Communications Technology, Kobe, Japan

**Keywords:** ciliated protozoa, protein kinase, heterologous gene expression, GFP-fusion proteins, nuclear and ciliary protein kinase, ICK-related kinase, MAK-related kinase

## Abstract

In the ciliate *Euplotes raikovi*, a 631-amino acid *Er*-MAPK1 protein kinase was found to localize in nucleoli of the transcriptionally active nucleus (macronucleus) and act as a key component of an autocrine, cell-growth promoting self-signaling mechanism. While its 283-amino acid N-terminal domain includes all the structural specificities of the mitogen-activated protein kinases required for a catalytic function, the 348-amino acid C-terminal domain is structurally unique with undetermined functions. By expressing the two *Er*-MAPK1 domains tagged with the green fluorescent protein in mammalian fibroblasts, the yeast *Schizosaccharomyces pombe* and the ciliate *Tetrahymena thermophila*, evidence was obtained that the C-terminal domain contains all the sequence information responsible for the *Er*-MAPK1 subcellular localization. However, in fibroblasts and *S. pombe* this information determined a nucleolar localization of the GFP-tagged C-terminal domain, and a ciliary localization in *T. thermophila*. In the light of these findings, the *Er*-MAPK1 localization in *E. raikovi* was re-examined via immunoreactions and shown to be ciliary besides that nuclear, as is the case for the mammalian intestinal cell kinase with which the *Er*-MAPK1 N-terminal domain shares a strong sequence identity and a catalytic function.

## Introduction

Protist ciliates possess remarkably high numbers of genes encoding protein kinases. The genomes of *Tetrahymena thermophila*, *Paramecium tetraurelia*, and *Stentor coeruleus* contain 1069, 2606, and 2057 kinase-coding genes, respectively, representing 4, 6.6, and 6% of the whole gene number (Eisen et al., 2006; Bemm et al., 2009; Reiff and Marshall, 2017). In man, in contrast, the 518 kinase-coding genes approximate to only 1.7% of all genes (Manning et al., 2002). However, which subcellular localization and function distinguish the products encoded by this multitude of ciliate kinase-coding genes are practically unexplored questions.

In the transcriptionally active somatic nucleus (macronucleus) of a free-living marine ciliate, *Euplotes raikovi*, a 631-amino acid protein kinase, designated *Er*-MAPK1, was identified in functional relation with the cell switching between the asexual and sexual stages of the life cycle, it appearing phosphorylated in vegetative cells growing stimulated by autocrine (autologous) interactions with their secreted (self) water-borne signaling protein pheromone and de-phosphorylated in cells temporarily induced to unite in mating pairs by competitive paracrine (heterologous) interactions with a non-self pheromone (Vallesi et al., 2010).

The site of the *Er*-MAPK1 enzymatic activity was clearly identified with the 283-amino acid N-terminal domain. Its sequence carries all the sub-domains that are common to serine/threonine protein kinases and, located in the activation loop, the Thr-Xxx-Tyr motif which is distinctive of the Mitogen-Activated Protein Kinases (MAPKs) widely conserved in eukaryotic cells and involved in regulating an ample variety of cellular processes including proliferation, differentiation, motility, stress response, and apoptosis (Plotnikov et al., 2011).

Differently from the *Er*-MAPK1 N-terminal domain, the 348-amino acid C-terminal domain appeared to be structurally unique and this uniqueness raised the question of which functions it plays. A function in driving the *Er*-MAPK1 nuclear localization was inferred by observing its extremely basic amino acid composition (isoelectric point, 10.2).

Considering a persistent lack of a reliable procedure to introduce, maintain, and faithfully express a modified chromosome into *Euplotes* cells, the above hypothesis was assessed by linking the Green-Fluorescent-Protein (GFP) to the *Er*-MAPK1 N- and C-terminal domains, and expressing the two tagged domains in three distinct heterologous systems represented by mammalian fibroblasts, the yeast *Schizosaccharomyces pombe* and the ciliate *Tetrahymena thermophila*. In addition to obtaining convergent evidence that the information for the *Er*-MAPK1 subcellular localization resides exclusively in the protein C-terminal domain, the expression in *Tetrahymena* revealed that this information may determine a ciliary beside that a nuclear localization.

## Materials and Methods

### Analysis of Protein Structure and Prediction of Nuclear Localization Signals (NLSs)

Protein sequence analysis and NLS prediction were performed *in silico*, using the internet website interfaces provided by the following prediction programs: InterPro: http://www.ebi.ac.uk/interpro/ (Mitchell et al., 2019); DICHOT: http://www.ideal.force.cs.is.nagoya-u.ac.jp/dichot/ (Fukuchi et al., 2011); PSORT II: https://psort.hgc.jp/form2.html (Nakai and Horton, 1999); Eukaryotic Linear Motif resources (ELM): http://elm.eu.org/search.html (Gouw et al., 2018); NLSmapper: http://nls-mapper.iab.keio.ac.jp/cgi-bin/NLS_Mapper_form.cgi (Kosugi et al., 2009).

### Amplification and Construction of Expression Vectors

The full-length *Er*-MAPK1 sequence and segments encoding the *Er*-MAPK1 N- and C-terminal domains were amplified from the *Er*-MAPK1 gene cloned into the pCR2.1-TOPO vector (Invitrogen) as previously described (Vallesi et al., 2010). No TGA stop codon (specifying cysteine in *Euplotes*) is present in the *Er*-MAPK1 gene coding region, and the +1-frameshifting site was removed by single-base deletion. Primers used in PCR amplifications (Table 1) were designed in such a way to add a restriction site to the 5′ end. Fragments amplified with primers containing *Bam*HI and *Xba*I restriction sites were cloned into the pVAX1 vector (Invitrogen), provided with both a human cytomegalovirus immediate-early promoter for a high-level protein expression and a bovine growth hormone polyadenylation signal for an efficient mRNA transcription termination. Fragments amplified with primers with *Sma*I restriction site into the thiamine-repressible pREP41 vector (Maundrell, 1993), and fragments amplified with primers with *Xho*I and *Apa*I restriction sites into the ribosomal DNA-based vector pIGF1 under the cadmium-inducible *MTT1* gene promoter (Shang et al., 2002; Malone et al., 2005). The correctness of constructs in each generated expression vector was confirmed by DNA sequencing.

**TABLE 1:** Primers used in this study.

**Gene sequence**	**Forward primer (5′–3′)**	**Reverse primer (5′–3′)**
GFP	ATGGCTAGCAAAG GAGAAGAACTC	GTTGTACAGTTC ATCCATGCC
N-terminal domain	ATGGACAGGTACA AGATAATC	TTAGAAGTAGTCA TGCTCCAGCAA
C-terminal domain	TTAAAATTTTGACC CATATCT	AAAGATTTTGTG CCGCCAGTC

### Transfection of Mammalian Fibroblasts

Fibroblasts of the NIH3T3 cell line were cultured in Dulbecco’s modified Eagle’s medium (DMEM) with GlutaMax-I supplemented with 10% fetal bovine serum (Invitrogen), 100 U/ml penicillin and 100 μg/ml streptomycin, and split every 2–4 days to maintain monolayer coverage. Twenty-four hours before transfection, 250,000 cells were seeded onto a sterile glass coverslips in 12-well culture plates, using medium without antibiotics and incubated in order to reach 70–80% confluence. Lipoplexes used for transfection were prepared in Opti-MEM I Reduced Serum Medium (Invitrogen) by mixing 2 μg of plasmid with 4 μl of Lipofectamine 2000 Reagent (Invitrogen) for each well. Cells were incubated in Opti-MEM for 4 h, then washed with phosphate buffer saline (PBS: 130 mM NaCl, 2 mM KCl, 8 mM Na_2_HPO_4_, 2 mM KH_2_PO_4_, pH 7.2) before being incubated in growth medium for 48 h. Transfected fibroblasts were fixed with 4% paraformaldehyde in PBS for 10 min at room temperature, and incubated with DAPI (Sigma-Aldrich) to visualize the nuclei. Coverslips were then mounted with Mowiol 4-88 (Calbiochem) onto microscope slides and observed on Nikon inverted microscope (Diaphot-TMD) connected to a laser-scanning confocal imaging system (MRC 600, Bio-Rad).

### Transfection of *S. pomb*e

*Schizosaccharomyces pombe* cells of strain TGO509 (*h^90^ leu1 gar2-mCherry:kanR*) were transfected as previously described (Moreno et al., 1991). This strain is auxotrophic for leucine, and transfection with pREP41-based GFP-*Er*-MAPK1 expression vectors can complement this leucine auxotrophy. Therefore, transfected cells were selected on solid minimal medium (EMM2) lacking leucine and supplemented with 2 μM thiamine to repress GFP-*Er*-MAPK1 constructs expression, and cultured in EMM2 without thiamine to induce GFP-*Er*-MAPK1 constructs expression. Living cells were then pressed between two coverslips and observed using a fluorescence microscope IX-70 equipping a PlanApo/60x/NA1.4 oil SC objective (Olympus, Tokyo, Japan). Fluorescence images were obtained by the DeltaVision system (Applied Precision, Issaquah, WA, United States).

### Transfection of *T. thermophil*a

Conjugating pairs between cell strains CU427 [*chx1-1*/*chx1-1* (CHX1; cy-s, VI)] and CU428 [*mpr1-1*/*mpr1-1* (MPR1; mp-s, VII)] were transfected as previously described (Iwamoto et al., 2014). Transfected cells were diluted into culture medium composed of 1.5% (w/v) proteose-peptone, 0.5% (w/v) yeast extract, and 0.5% (w/v) D-glucose (Difco) and aliquoted to 96-wells plates. After incubation at 30°C for 18 h, paromomycin sulfate (Duchefa Biochemie) was added as a selective drug at the final concentration of 120 μg/ml. Successfully transformed cells exhibiting paromomycin resistance grew after 3 days of selective cultivation. CdCl_2_ at concentrations of 1.0–2.0 μg/ml was added to the culture in logarithmic growth phase at approximately 3 h before observation. Living cells were observed using a fluorescence microscope IX-70 equipping a UApo/40x/NA1.35 objective (Olympus, Tokyo, Japan).

### Immunofluorescence Analysis

Immunofluorescence analysis of transfected fibroblasts was performed to visualize nucleoli, using anti-fibrillarin antibodies (Thermo-Fisher). Briefly, cells were fixed with 4% paraformaldehyde, permeabilized with 0.2% Triton-X-100 in PBS for 5 min, blocked for 30 min in 1% BSA in PBS, and incubated for 1 h with the primary antibody, followed by incubation for 1 h with the DyLight 594-conjugated anti-mouse antibodies (Bethyl Laboratories, Inc.). Samples were washed with PBS, then mounted with Mowiol 4-88 (Calbiochem) onto microscope slides for observation.

To analyze *Er*-MAPK1 ciliary localization, *E. raikovi* cells were incubated with Triton-X in microtubule-stabilizing buffer (60 mM PIPES, 25 mM HEPES, 10 mM EGTA, 2 mM MgCl_2_, pH 6.9), as previously described (Kloetzel et al., 2003). Cells were then fixed on slides with 80% ethanol, rehydrated in PBS and incubated at room temperature for 1 h with 1% bovine serum albumin (BSA) and a second hour with a custom-synthesized antibodies (anti-S_489_-R_502_) (GenScript) directed against the sequence Ser_489_-Arg-Lys-Asp-Ser-Arg-Lys-Glu-Ser-Arg-Lys-Asp-Ser-Arg_502_ which is exclusive of the *Er*-MAPK1 C-terminal domain. After washing with PBS, samples were finally incubated for 1 h with DyLight 488-conjugated anti-rabbit antibodies (Bethyl Laboratories, Inc.) washed again, and mounted onto microscope slides for observation.

## Results

### *Er*-MAPK1 Structure and Putative NLSs

**FIGURE 1:**
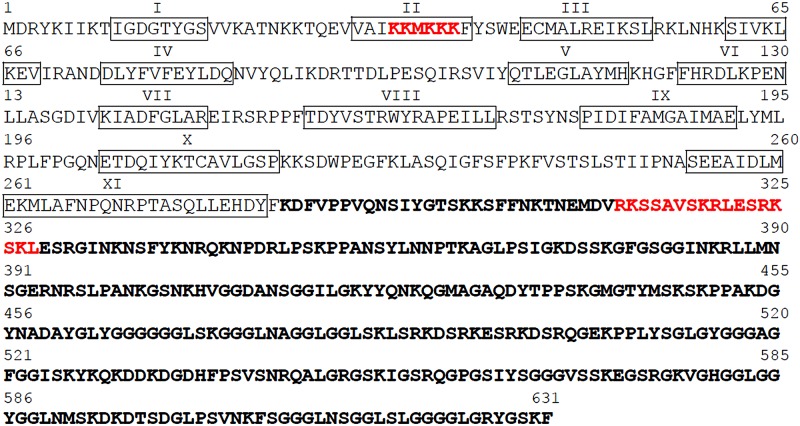
Amino acid sequence of *Er*-MAPK1. The N- and C-terminal domains are written with regular and bold letters, respectively. The eleven MAPK-specific sub-domains are boxed and indicated by roman numbers; predicted NLS sequences by the programs PSORT II (score, 0.51), ELM motif search (probability, 2.588 e^–04^), and NLSmapper (score 4.7) are written in red.

Amino acid sequence and structural motifs of *Er*-MAPK1 are shown in Figure 1. Two different protein domains are clearly identified by *in silico* analyses. The Met_1_ to Phe_283_ N-terminal domain contains the eleven sub-domains for the kinase catalytic activity. The Lys_284_ to Phe_631_ C-terminal domain is predicted to have an intrinsically disordered configuration, a structural feature which is considered suitable for the interaction with other proteins by exposing short linear peptide motifs (Babu et al., 2012; van der Lee et al., 2014). Commonly used web-based prediction programs recognized two distinct NLS motifs. One lies in the protein N-terminal domain and is represented by the hexapeptide Lys_33_-Lys-Met-Lys-Lys-Lys_38_ matching the general consensus sequence Lys(Lys/Arg)Xxx(Lys/Arg) of mono-partite NLS motifs (Lange et al., 2007). It is internal to sub-domain II responsible for the phosphor-transfer activity of the kinase. A second predicted motif, Arg_312_-Lys-Ser-Ser-Ala-Val-Ser-Lys-Arg-Leu-Glu-Ser-Arg-Lys-Ser-Lys-Leu_328_, resides in the *Er*-MAPK1 C-terminal region and is recognized as a bipartite variant of the classical basically charged NLS (Dingwall and Laskey, 1991; Robbins et al., 1991). It is formed by two basic residues a 10-residue spacer, and another basic region consisting of three basic residues out of five residues.

### Expression in Fibroblasts

**FIGURE 2:**
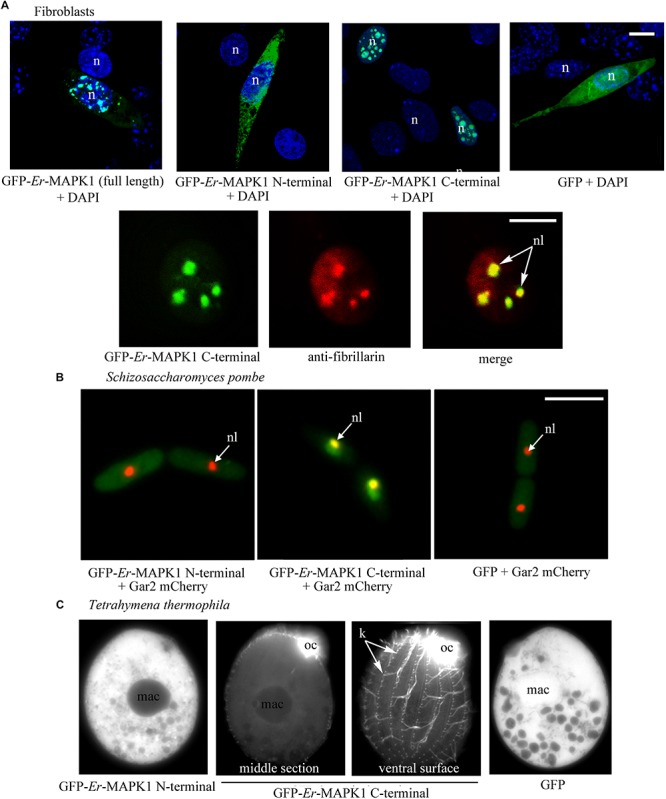
Heterologous expression of GFP-tagged *Er*-MAPK1 domains. **(A)** Top: representative fluorescence images of transfected fibroblasts (green). Nuclei are stained with DAPI (blue). GFP-*Er*-MAPK1 full-length and GFP-C-terminal proteins localize into the nucleus, and the GFP-N-terminal protein in the cytoplasm. GFP alone, used as control, diffuses in both cytoplasm and nucleus due to its molecular weight (28 kDa) below the exclusion size of nuclear pores. Bottom: magnification of a fibroblast nucleus in which the GFP-C-terminal protein (green) and anti-fibrillarin antibodies (red) co-localize in nucleoli (yellow). Each image represents a single section. **(B)** Representative fluorescence images of *S. pombe* cells co-expressing the GFP-N-terminal protein, the GFP-C-terminal protein and GFP alone (green) with the nucleolar protein Gar2 (red). The nucleolar co-localization (central picture) between the GFP-C-terminal protein and the Gar2 protein is yellow. **(C)** Representative fluorescence images of *T. thermophila* expressing the GFP-N-terminal protein, the GFP-C-terminal protein and GFP alone. The GFP-C-terminal protein accumulates in the oral ciliature and single somatic cilia. n, nucleus; nl, nucleolus; mac, macronucleus; oc, oral ciliature; k, kinetosomes in a ciliary row; bars, 10 μm.

In murine fibroblasts of the NIH3T3 line, the individual expression of the *Er-*MAPK1 N-terminal and C-terminal domains was carried out in parallel with the expression of the full-length *Er-*MAPK1 sequence in such a way to preliminarily verify the capability of a heterologous system to accomplish a correct *Er-*MAPK1 nuclear localization. The transfection was run by ligating the coding sequences of the three proteins downstream and in-frame with the GFP-coding gene, and inserting the ligated sequences into the mammalian expression vector pVAX. Analyzed in confocal microscopy (Figure 2A), transfected fibroblasts revealed an equal spotted nuclear localization for both the entire *Er*-MAPK1 protein and the C-terminal domain, and the spots were shown to be nucleoli by proving co-localization between the GFP signal of the fusion proteins and the immune-staining generated by fibrillarin antibodies used as nucleolar marker. In contrast, the N-terminal fusion protein appeared uniformly dispersed throughout the cell cytoplasm, implying that no specific sequence motif of the *Er*-MAPK1 N-terminal domain, and the predicted NLS in particular, are involved in the protein nuclear localization.

### Expression in *S. pomb*e

The expression of the GFP-*Er*-MAPK1 constructs in *S. pombe* was performed in cells synthesizing the nucleolar Gar2 protein tagged with the red fluorescent protein mCherry (Gulli et al., 1995), using construct copies amplified via PCR from the vector pVAX1 and ligated into the multi-cloning site of vector pREP41 (Maundrell, 1993). Transfected *S. pombe* cells were analyzed in fluorescence microscopy after having been cultivated for 15 h in absence of thiamine to induce protein synthesis. Fully matching the transfected fibroblasts, they showed (Figure 2B) the GFP-C-terminal protein concentrated inside the nucleus and, in contrast, the GFP-N-terminal protein uniformly dispersed throughout the cell cytoplasm. The GFP-C-terminal protein association to nucleoli was in this case validated by showing co-localization between the GFP signal and the mCherry fluorescence of the Gar2 protein.

### Expression in *T. thermophila* and *Er*-MAPK1 Immunolocalization in *E. raikov*i

For the expression in *T. thermophila*, the GFP-*Er*-MAPK1 constructs were inserted into the vector–pIGF1, and cells were analyzed under fluorescence microscope 3 days after transfection and exposure to CdCl_2_. Alike fibroblasts and *S. pombe*, transfected *T. thermophila* cells revealed a specific subcellular localization only of the GFP-C-terminal protein. However, this localization was ciliary, not nucleolar (Figure 2C). The fluorescent signal revealed a particularly intense association to the kinetosomes of the somatic single cilia covering the cell body surface, and produced a massive (unresolved) staining at level of the oral membranelles surrounding numerous the cell cytostome.

**FIGURE 3:**
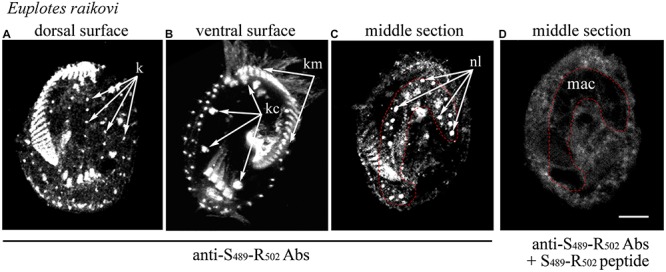
Localization of *Er*-MAPK1 in *E. raikovi*. Representative images of cells incubated with antibodies directed against the sequence Ser_489_-Arg_502_ of the C-terminal domain **(A–C)**, and a mixture between the same antibodies and the Ser_489_-Arg_502_ peptide **(D)**. The position of the macronucleus is outlined by a red dotted line. k, kinetosomes of dorsal cilia (bristles); kc and km, packed kinetosomes of cirri and membranelles, respectively; nl, nucleolus; mac, macronucleus; bar, 10 μm.

In the light of this ciliary localization of the GFP-C-terminal protein in *T. thermophila*, the native localization of the entire *Er*-MAPK1 protein was re-examined in *E. raikovi* via immunoreactions carried out with antibodies generated against a strong immunogenic, *Er*-MAPK1-specific sequence, Ser_489_-Arg_502_, which lies centrally in the C-terminal domain and finds no significant counterpart in other proteins. As shown in Figure 3, in addition to decorating nucleoli in the cell macronucleus, these antibodies bound intensely also to the kinetosomes of the whole (somatic and oral) *E. raikovi* ciliature, represented by the single bristle cilia on the cell dorsal surface and compound ciliary organelles (cirri and adoral membranelles) on the ventral surface. From these immunoreactions it was thus evident that the *Er*-MAPK1 subcellular localization in *E. raikovi* is not exclusively nuclear, but also ciliary.

## Discussion

By heterologous expression of GFP-tagged N- and C-terminal proteins in mammalian fibroblasts, *S. pombe* and *T. thermophila*, we provided evidence that (i) the subcellular localization of the *E. raikovi Er*-MAPK1 protein kinase depends on sequence information residing exclusively in the structurally unique, extremely basic C-terminal domain, and (ii) this domain may target the protein to the nucleus in specific association with nucleoli as originally reported in the native organism, as well as to cilia in apparently preferential association with the kinetosomes.

To transit into the nucleus and cilia, proteins rely on NLS sequences which are recognized and bound by importins that govern their passage through nuclear pore complexes and ciliary gates (Dishinger et al., 2010; Kee et al., 2012; Kee and Verhey, 2013; Bauer et al., 2015; Del Viso et al., 2016). The Arg_312_^_^Leu_328_ sequence identified in the *Er*-MAPK1 C-terminal domain as NLS by predictive programs presents all the requisites to act as key site of interactions with importins. It contains two clusters of basic amino acids separated by a short spacer region as classical bipartite NLSs, and is well exposed on the molecular surface, hence capable to interact with transporter proteins. In addition, it appears to be closely conserved among *Er*-MAPK1 homologs of other *Euplotes* species such as *E. vannus* (Chen et al., 2019), *E. nobilii* (Candelori et al., 2013) and *E. petzi* (unpublished results), as well as among *Er*-MAPK1 analogs characterized from other ciliates. The conclusion that the Arg_312_^_^Leu_328_ sequence is directly responsible for the *Er*-MAPK1 subcellular localization, is not necessarily weakened by the observation that *T. thermophila* localized the *Er*-MAPK1 GFP-C-terminal protein to cilia, and not to the nucleus. *T. thermophila* is known to have a peculiar mechanism of nuclear entry, with several “universal” NLSs that are not recognized by transporter proteins and *Tetrahymena* NLSs that have no function in other eukaryotic cells (Iwamoto et al., 2018).

Among serine/threonine kinases more closely comparable to *Er*-MAPK1 in amino acid sequence, more significant relationships were found to link *Er*-MAPK1 to the flagellar kinase MAPK7 identified from the *Chlamydomonas* genome (Merchant et al., 2007), and the mammalian Male-germ cell Associated Kinases (MAKs) and Intestinal Cell Kinases (ICKs), initially identified in relation to mechanisms underlying the proliferative and developmental cell response to growth factors (Togawa et al., 2000; Ma et al., 2006; Fu et al., 2009), and later detected also in cilia where they act as negative regulators of the ciliary length and ciliogenesis (Chaya et al., 2014; Moon et al., 2014; Oud et al., 2016). In a functional perspective, however, the *Er*-MAPK1 relationships with mammalian ICKs and MAKs appeared to be much more meaningful than the relationships with MAPK7, considering that this kinase has an extra-nuclear localization and an amino acid sequence that, lacking a distinct C-terminal domain, extends only half of the *Er*-MAPK1 sequence. This conclusion is here reinforced by the experimental evidence that *Er*-MAPK1, in addition to showing the same double phosphorylation Thr-Asp-Tyr site as ICKs and MAKs and a matching sequence length and organization, shares also a double, nuclear and ciliary, subcellular localization, which suggests to credit this *Euplotes* kinase with an early evolved multiple, context-dependent activity.

## Data Availability Statement

All datasets generated for this study are included in the manuscript/supplementary files.

## Author Contributions

AC, AA, and AV conceived and planned the study. AC, TY, MI, and MM performed the experiments and analyzed the data. AC, TY, and MI prepared the figures. AV wrote the manuscript with the contribution of all authors.

## Conflict of Interest

The authors declare that the research was conducted in the absence of any commercial or financial relationships that could be construed as a potential conflict of interest.

The reviewer EV declared a past co-authorship with one of the authors AV to the handling Editor.
